# Neuropsychiatric Manifestations of COVID-19 Disease and Post COVID Syndrome: The Role of N-acetylcysteine and Acetyl-L-carnitine

**DOI:** 10.2174/011570159X343115241030094848

**Published:** 2024-11-05

**Authors:** Tommaso Barlattani, Giuseppe Celenza, Alessandro Cavatassi, Franco Minutillo, Valentina Socci, Carolina Pinci, Riccardo Santini, Francesca Pacitti

**Affiliations:** 1 Department of Biotechnological and Applied Clinical Sciences, University of L’Aquila, 67100 L’Aquila, Italy;; 2 Department of Systems Medicine, University of Rome Tor Vergata, Rome, Italy

**Keywords:** COVID-19, SARS CoV-2, post-acute COVID-19 syndrome, long COVID brain fog, acetylcysteine, acetylcarnitine, SLC7A11 protein, metabotropic glutamate receptor 2, metabotropic glutamate receptor 3

## Abstract

COVID-19 is associated with neuropsychiatric symptoms, such as anosmia, anxiety, depression, stress-related reactions, and psychoses. The illness can cause persistent cognitive impairment and “brain fog”, suggesting chronic brain involvement. Clinical entities of ongoing symptomatic COVID-19 and Post COVID Syndrome (PCS) mainly present neuropsychiatric symptoms such as dysgeusia, headache, fatigue, anxiety, depression, sleep disturbances, and post-traumatic stress disorder. The pathophysiology of COVID-19-related brain damage is unclear, but it is linked to various mechanisms such as inflammation, oxidative stress, immune dysregulation, impaired glutamate homeostasis, glial and glymphatic damage, and hippocampal degeneration. Noteworthy is that the metabotropic receptor mGluR2 was discovered as a mechanism of internalisation of SARS-CoV-2 in Central Nervous System (CNS) cells. N-acetylcysteine (NAC) and acetyl-L-carnitine (ALC) are two supplements that have already been found effective in treating psychiatric conditions. Furthermore, NAC showed evidence in relieving cognitive symptomatology in PCS, and ALC was found effective in treating depressive symptomatology of PCS. The overlapping effects on the glutamatergic system of ALC and NAC could help treat COVID-19 psychiatric symptoms and PCS, acting through different mechanisms on the xc-mGluR2 network, with potentially synergistic effects on chronic pain and neuro-astrocyte protection. This paper aims to summarise the current evidence on the potential therapeutic role of NAC and ALC, providing an overview of the underlying molecular mechanisms and pathophysiology. It proposes a pathophysiological model explaining the effectiveness of NAC and ALC in treating COVID-19-related neuropsychiatric symptoms.

## 1 INTRODUCTION

The global impact of the COVID-19 pandemic, including its repercussions in terms of infections and casualties within the population, has garnered significant worldwide interest [1].

SARS-CoV-2 infection primarily targets the pulmonary system, usually initiating pneumonia and potentially life-threatening respiratory damage [1]. SARS-CoV-2 infection induces damage and detrimental effects on endothelial cells, instigating a cascade of events, including inflammation, thrombosis, and cerebral damage [2].

Therefore, it is noteworthy that neuropsychiatric manifestations of COVID-19 are prevalent [3]. Although the variants have changed [4], there is still potential for neuropsychiatric sequelae, and a large cohort study identified an equal potential of neuropsychiatric consequences comparing omicron *versus* delta variants [5]. When delving into the neuropsychiatric sequelae of COVID-19 disease, it is imperative to encompass both the ongoing symptomatic COVID-19 and the PCS [6]. The National Institute for Health and Care Excellence (NICE) defines these clinical entities as signs and symptoms during or after COVID-19 infection [7]. These symptoms persist for a period ranging from 4 to 12 weeks in the case of ongoing symptomatic COVID-19 and extend beyond 12 weeks in the context of PCS, provided that a clear alternative diagnostic explanation is not present [7]. In the context of ongoing symptomatic COVID-19 and PCS, a spectrum of neurological and neuropsychological symptoms have been documented [8, 9]. In particular, among the array of psychiatric and cognitive symptoms, the most commonly represented are depression, anxiety, fatigue, “brain fog”, sleep disturbances, and PTSD [8, 10, 11]. Moreover, an elevated propensity for suicidal behaviour was outlined [12].

The underlying mechanisms responsible for the pathophysiology of acute and persistent neuropsychiatric symptoms in COVID-19 and PCS remain not completely elucidated [8,13]. Previous investigations into systemic viral infections have proposed a pathogenic hypothesis centered on a sustained neuroinflammatory response to viral antigens and the mobilisation of proinflammatory mediators and immune cells from the peripheral circulation [14]. Despite the etiopathological uncertainties, several mechanisms have been postulated [15]. Aberrant inflammation is among these [16], associated with other factors, including oxidative stress induced by Reactive Oxygen Species (ROS), glial and astrocyte injury, compromised monoamine secretion, dysregulation of glutamatergic pathways, neuro excitotoxicity, damage to cerebral blood vessels [15, 17], dysfunction in glymphatic processes [18], and disruption of hippocampal function and dorsolateral prefrontal cortex (DLPFC) [19]. The role of the mGluR2 brain receptor is crucial, as it plays a pivotal role in the internalisation process of SARS-CoV-2 within cells [20], given its association with various psychiatric disorders [21, 22].

Considering the nature of the neuropsychiatric manifestations, compounds able to modulate the underlying molecular mechanisms and biochemical pathways can be considered. For these purposes, N-acetylcysteine (NAC) and acetyl-L-carnitine (ALC), even in consideration of their molecular target and pleiotropic effect, are valuable therapeutic options.

For instance, the overlapping effects of ALC and NAC on the glutamatergic system could act through different mechanisms on the xc-mGluR2 network, which may synergistically affect chronic pain and neuro-astrocyte protection.

Noteworthy, these compounds stand out as effective in treating various psychiatric conditions.

It is not by chance that NAC has been shown to alleviate cognitive symptoms in PCS [23], while ALC has been effective in treating depressive symptoms of PCS [24].

Thus, within this pathological framework and considering their multifaceted pharmacological profile, NAC and ALC look promising for mitigating COVID-19-associated neuropsychiatric symptoms and PCS [24-28]. Considering the absence of specific guidelines on effective treatment for the management of neuropsychiatric symptoms of acute COVID-19 and PCS, there is considerable interest in identifying possible candidate drugs.

In attempting to describe and hypothesis the mechanisms underlying the success of NAC and ALC in mitigating the neuropsychiatric symptoms associated with COVID-19 and PCS, the authors present a comprehensive and detailed overview of both molecular and pathophysiological pivotal mechanisms, proposing a potential pathophysiological model accounting for NAC and ALC effectiveness in treating COVID-19-related neuropsychiatric symptoms. To define and speculate on the molecular mechanism, this article will first summarise the mechanisms of entry of SARS-CoV-2 and its consequences in the CNS and related symptoms at the molecular level. Finally, the known mechanisms of action of NAC and ALC will be outlined in order to hypothesise a rationale for the use of NAC and ALC as a treatment in the outlined conditions.

## 2 LITERATURE REVIEW

This narrative review was conducted using the following search terms: “COVID-19”; “Sars CoV-2”; Post-COVID Syndrome”; “PCS”; “Post-acute COVID syndrome”; “PACS”; “neuropsychiatric symptoms” “N-Acetyl Cysteine”; “NAC”; “Acetyl-L-Carnitine”; “ALC”; “Metabotropic Glutamate receptor”; “mGlu”; “mGluR2”; “mGluR3”; “Metabotropic receptors”; Cystine-glutamate antiporter”; “Xct”; “Nuclear factor erythroid 2-related factor”; “Nrf2”; “Nuclear factor Kb”; “Nf-kB”; “Blood-Brain Barrier”; “BBB”; “Central Nervous System”; “CNS”; “Blood-Cerebrospinal Fluid Barrier”; “BCB”; “Interleukin”; “IL; “Tumoral Necrosis Factor”; “TNF”; “angiotensin-converting enzyme 2 receptor”; “ACE2”; “Transferrin receptor”; “TfR1”; “interferon”; “IFN”, “monocyte chemoattractant protein”; “MCP”; reactive oxigen species; ROS “reactive nitrogen species”; “RNS”, “glial fibrillary acidic protein”; “GFAP”; “Excitatory amino-acid Transporter 2”; “EAAT2”; “N-methyl-D-aspartate receptor”; “NMDA”; “Chronic Fatigue Syndrome”; “CFS”; “Dorso-Lateral prefrontal cortex”; “DLPFC”; “kynurenic acid”; “KYNA”; “Nf-kB essential modulator”; “NEMO”; were entered in ERIC, MEDLINE, PsycARTICLES, PsycINFO, Scopus and PubMed.

Terms and databases were combined using the Boolean search technique to make search more restrictive and detailed. Only studies published in English have been included. The main results were then discussed narratively divided by paragraphs, respectively: SARS-CoV-2 Neuroinvasion in COVID-19: overview of Entry Mechanisms; COVID-19-induced Brain Damage and Neuropsychiatric Consequences; Neuropsychiatric Symptoms in Acute COVID-19, Ongoing COVID-19, and PCS; NAC and ALC: overview and mechanism of action; Hypothesis and Rationale of NAC and ALC in neuropsychiatric manifestations of acute COVID-19 disease, ongoing symptomatic COVID-19, PCS”.

## 3 SARS-CoV-2 NEUROINVASION IN COVID-19: OVERVIEW OF ENTRY MECHANISMS

**Fig. (1):**
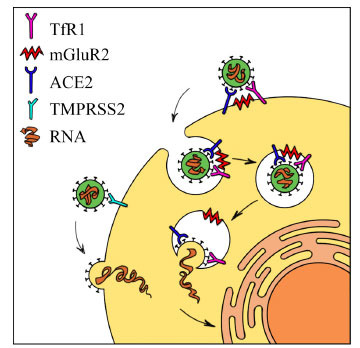
Potential mechanism of SARS-CoV-2 internalisation. **Abbreviations:** TfR1: Transferrin Receptor type 1; mGluR2: metabotropic Glutamate 2 receptor; ACE2: angiotensin-converting enzyme 2 receptor; TMPRSS2: type II Transmembrane Serine Proteases; RNA: Sars-Cov-2 virus RNA.

SARS-CoV-2 directly affects the nervous system [29]. Many theories tried to explain how the virus enters the brain and its impact on it [2]. One is that the olfactory tract represents the preferential way through the axons from the cribriform plate, explaining the loss of smell associated with COVID-19 [30]. Another theory is that the virus enters the brain *via* the vagal and trigeminal nerves [31]. It is also suggested that the virus may enter the brain through the paranasal lymph vessels and circumventricular organs (CVOs) [32] (Fig. **1**).

One of the most extensively studied pathways for SARS-CoV-2 infection involves host entry through the angiotensin-converting enzyme 2 (ACE2) receptor [33]. This receptor is expressed in endothelial cells within the central nervous system (CNS) [34], in neurons and glial cells [35], as well as in the choroid plexus [36]. A study by Wang *et al*. in 2021 underlined the role of mGluR2 participation in the mechanism of COVID-19 infection and cellular internalisation [23]. The mGluR2 itself plays an inhibitory role by depleting cyclic Adenosine Mono Phosphate (cAMP) and consequently facilitating the opening of K^+^ channels, thus preventing neuro-excitotoxicity [37]. It works as a co-receptor through a clathrin-mediated mechanism, thereby favouring the process of virus internalisation [38]. mGluR2 is widely expressed in the CNS, both in neurons and glia [39], and it is involved in the pathophysiology of epilepsy, dementia, autism, and various psychiatric illnesses [21, 22, 40]. Specifically, it has been observed that Transferrin receptor 1 (TfR1) interacts with mGluR2, leading to their co-internalization within the same clathrin-coated pit, ultimately facilitating the entry of Coronaviruses [38]. Furthermore, it is noteworthy that TfR1 directly binds to the SARS-CoV-2 spike protein [38].

Cortical astrocytes appear particularly susceptible to SARS-CoV-2 infection [41], impairing protein folding, translation initiation, and metabolic functions. Together with astrocytes, pericytes also tend to be infected, resulting in impaired functioning due to receptor blockade and capillary constriction [42]. Even brain epithelial cells are infected through the cleavage of the S2 subunit of the TMPRSS-2, a transmembrane protease expressed in these cellular types, thus allowing the entry of SARS-CoV-2 [43] (Fig. **1**).

Moreover, SARS-CoV-2 infection may damage the choroid plexus epithelium, leading to leakage of immune cells and cytokines into the cerebrospinal fluid (CSF) [36, 44]. The discovery of the glymphatic system, the brain-wide network of perivascular channels with a role in CSF recirculation [45], opens up new possibilities for understanding how COVID-19 damages the brain [18]. Notably, a glymphatic dysfunction was found in subjects recovered from mild COVID-19 [46]. The engagement of this network aligns with the CSF profile observations in COVID-19 patients exhibiting neurological symptoms and experiencing long COVID. These individuals typically demonstrate cerebrospinal epitheliopathy, blood-CSF barrier (BCB) dysfunction, and elevated cytokine levels [44]. Astrocytic endfeet contribute to the regulation of microvessel dilation and constriction, thereby exerting control over blood flow [47, 48], underscoring the dependence of the glymphatic system functioning on the optimal functioning of astrocytes [49, 50].

The receptors involved in SARS-CoV-2 infection are numerous and still under investigation [43], but ACE2 appears to be the primary receptor facilitating viral entry. Astrocytes express ACE2 [35], CD147, and DPP4 receptors, all of which supposedly serve as co-receptors for viral internalisation [41]. Consequently, alterations in the astrocytic and glymphatic system’s function are intricately linked to the neuropathological manifestations observed in COVID-19 cases. Furthermore, it is noteworthy that astrocytes, in concert with the choroid plexus, nasal lymphatic vessels, and CSF, are recognised as integral constituents of the glymphatic system thereby, the glymphatic system may provide a conduit for viral infiltration into the brain [18].

In conclusion, SARS-CoV-2 invades the brain through multiple pathways, including the olfactory tract, vagal and trigeminal nerves, and paranasal lymph vessels, with ACE2 being the primary receptor facilitating viral entry. Other receptors, such as mGluR2 and TfR1, also contribute to the virus's internalization, particularly affecting astrocytes and pericytes, leading to significant neurological dysfunction. The virus's impact on the glymphatic system and choroid plexus further exacerbates neurological symptoms, highlighting the intricate relationship between SARS-CoV-2 infection and CNS pathology. These findings underscore the complexity of COVID-19's effects on the nervous system and its long-term consequences.

## 4 COVID-19-INDUCED BRAIN DAMAGE AND NEUROPSYCHIATRIC CONSEQUENCES

The hallmark feature of COVID-19 disease is the severe cytokine storm, primarily responsible for acute respiratory distress syndrome (ARDS) and possible subsequent Multiple Organ Dysfunction Syndrome (MODS) [51, 52].

Patients with COVID-19 infection often exhibit elevated serum concentrations of pro-inflammatory cytokines, including interleukin (IL) IL-1, IL-6, IL-10, and tumor necrosis factor (TNF)-α [53]. Inflammatory markers such as IL-2, IL-6, IL-7, IL-10, IL-18, interferon (IFN)-γ, monocyte chemoattractant protein (MCP)-1, MCP-3, and macrophage-inflammatory protein-1α have been associated with symptom severity [54-56]. TNF-α can directly breach the BBB, leading to microglia and astrocyte activation [57, 58]. IL-6 and TNF-α levels are predictive of symptom severity and survival rates due to increased production of ROS and Reactive Nitrogen Species (RNS), with consequent oxidative stress-induced apoptosis [20, 54, 59]. Excessive ROS production also leads to mitochondrial dysfunction, DNA damage, and potential alterations in neurotransmitter signaling [60-62].

An epidemiological and clinical characteristics study revealed that 52% of COVID-affected patients exhibited increased IL-6 levels; among this cohort, 86% displayed markedly elevated C-reactive protein levels, indicating a substantial inflammatory response [2]. IL‐6 negatively modulates neural stem cell proliferation and cell fate in the hippocampus [63, 64]. Moreover, the Choroid Plexus can release the proinflammatory cytokines IL-6 and IL-8 into the CSF, thus augmenting the host's immune response [65].

In the CNS, microglia and astrocytes are the primary sources of IL-6 synthesis [66]. In physiological conditions, astrocytes play a crucial role in synthesising essential neurotransmitters like glutamate [67]. The responsible enzyme, glutamine synthetase, is primarily located within astroglial cells and converts glutamate into glutamine [68]. Astrocytes also regulate neuronal glutamate release using the xc- system, which facilitates the uptake of extracellular cysteine while releasing intracellular glutamate at a 1:1 ratio [69-71]. The significance of xCT is also linked to its role in GSH production, providing defense against oxidative stress-induced damage and its modulation of mGluRs [39, 72].

During inflammatory states, astrocytes can switch to an activated phenotype, characterised by heightened levels of glial fibrillary acidic protein (GFAP), which is a hallmark of activated astrocytes [73]; elevated GFAP levels have been observed in COVID-19 patients [74]. Indeed, post-mortem studies of COVID-19 patients have revealed the presence of activated microglia [75]. This has generated substantial interest in the role of microglial cells, which, in conjunction with astrocytes, play a crucial role in orchestrating the modulation of neuroinflammatory processes [76, 77]. When acute inflammation persists without resolution, it can lead to prolonged microglial activation. This sustained activation amplifies cytokine production and exacerbates oxidative stress damage [15]. Microglial cells are pivotal in initiating reactive astrogliosis through the Nf-κB signalling pathway and can induce glutamate release [78]. It also triggers the formation of ROS [79], which, in turn, damages healthy tissues, ultimately contributing to brain impairment [80]. The consequences of reactive astrogliosis and microglial activation following SARS-CoV-2 infection can initiate a cascade of aberrant signalling events, potentially leading to excitotoxicity which represents a pathological process with deleterious consequences [15, 35].

A study carried out by Fernández-Castañeda *et al*. (2022) utilising a murine model of mild SARS-CoV-2 infection localised to the respiratory system revealed several significant findings, including elevated cytokine levels in the CNS, impairment of hippocampal neurogenesis, and specific microglial reactivity observed in white matter regions [81]. Moreover, the increased and persistent inflammatory state caused by COVID-19 switches tryptophan metabolism to the kynurenine pathway instead of serotonin production [82]. Reactive astrogliosis and subsequent Nf-κB activation downregulate Excitatory amino-acid Transporter 2 (EAAT2), increasing extracellular glutamate levels [22]. The excess glutamate impairs xc- system activity, leading to heightened neuronal excitability, excessive activation of extrasynaptic N-methyl-D-aspartate (NMDA) receptors, and, in severe cases, excitotoxicity [83, 84]. This process is associated with reduced GSH levels, leading to increased production of ROS [85]. Various studies have shed light on the possibility that cognitive dysfunction related to COVID-19 may be attributed to transient damage to the DLPFC, hippocampal impairment, and disruptions in glutamate levels [19, 86-89].

Glutamate plays a crucial role in memory and learning processes, including long-term potentiation, which heavily relies on the hippocampus [90]. The hippocampus is rich in mGluR2 [91], and animal studies have shown that coronaviruses can induce neurodegeneration in the hippocampal CA1 and CA3 regions, leading to reduced short-term learning abilities and spatial memory impairment [92]. COVID-19-induced damage to hippocampal neurogenesis was also observed in humans [93]. In addition, DLPFC is also rich in mGluR2 [94, 95], and several pieces of evidence have shown that it is the most damaged by COVID-19, which can explain the cognitive dysfunction also observed in long COVID [28, 86]. The expression of mGluR2 serves as a critical link connecting COVID-19-induced brain damage, astrocyte damage, excitotoxicity, DLPFC, and hippocampal impairment (Ma *et al*., 2023; Scholler *et al*., 2017; Spampinato *et al*., 2018; Wang *et al*., 2021; X. Wang *et al*., 2023). Moreover, increased ACE2 expression in mice has been associated with enhanced sympathetic activity [96, 97] and reduced tryptophan uptake [98], which may increase susceptibility to the effects of stressful conditions [99]. Additionally, hypothalamic ACE2 receptors are implicated in the suppression of corticotropin-releasing hormone synthesis, which is a crucial component of the physiological stress response [96-101].

**Fig. (2):**
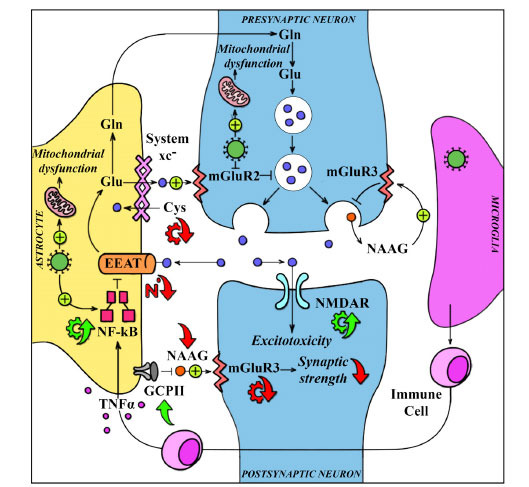
Potential pathophysiological mechanisms of SARS-CoV-2 on the CNS. **Abbreviations:** EAAT: excitatory amino acid transporter; NF-κB: Nuclear Factor-kappa B; NAAG: N-Acetyl-Aspartyl-Glutamate; mGluR3: metabotropic Glutamate 3 receptor; mGluR2: metabotropic Glutamate 2 receptor; Glu: Glutamate; Gln: Glutamine; Cys: Cysteine; System xc^-^: cystine/glutamate antiporter system; GCPII: Glutame Carboxypeptidase II; NMDAR: N-methyl-D-aspartate Receptor; TNFα: Tumor Necrosis Factor α.

In addition, recent findings have demonstrated that the SARS-CoV-2 metalloprotease cleaves Nf-κB essential modulators in brain endothelial cells [102]. This process leads to endothelial cell death, neuroinflammation, and the breakdown of BBB. Consequently, the compromised BBB allows the virus to breach the brain's protected environment while also permitting the entry of pro-inflammatory cytokines. This complex interplay fosters viral invasion and is pivotal in establishing a neuroinflammatory state [102, 103]. The convergence of evidence supports the premise that SARS-CoV-2 has a discernible impact on the CNS. This impact offers a plausible explanation for the acute neuropsychiatric symptoms observed in COVID-19 patients and long-term consequences (Fig. **2**).

In conclusion, COVID-19-induced brain damage and neuropsychiatric consequences stem from a severe cytokine storm that breaches the blood-brain barrier, leading to neuroinflammation, oxidative stress, and excitotoxicity. Elevated cytokines like IL-6 and TNF-α activate microglia and astrocytes, causing damage to critical brain areas such as the hippocampus and DLPFC, which are essential for memory and cognition. This neuroinflammatory state, along with disrupted neurotransmitter signaling, explains the acute and long-term neurological and cognitive symptoms observed in COVID-19 patients and long COVID cases.

## 5 NEUROPSYCHIATRIC SYMPTOMS IN ACUTE COVID-19, ONGOING COVID-19, AND PCS

As observed within SARS and MERS, SARS-CoV-2 is associated with delirium, depression, anxiety, post-traumatic consequences, memory impairment, insomnia, encephalopathy and encephalitis during the acute phase [3, 17]. A recent study using data from two independent retrospective cohort studies found delirium, anxiety, and mood symptoms as the most commonly represented in the acute phase of infection [104]. Moreover, migraine-like headaches refractory to traditional analgesics [105] were also reported during the ongoing infection [106]. In particular, headaches, together with anosmia and ageusia, may persist beyond the resolution of other symptoms and endure for up to 6 months following recovery from both acute and ongoing infection [107, 108].

Moreover, approximately 30-40% of patients who had contracted COVID-19 reported clinically significant levels of depression and anxiety [109, 110]. In a cohort of 402 COVID-19 survivors in Italy, approximately 56% of individuals screened positive one month after hospitalisation for at least one psychiatric domain [110]. In a comprehensive analysis of a real-world dataset comprising 62,354 COVID-19 survivors from 54 healthcare institutions in the USA [111], the estimated incidence of psychiatric disorders, both initial and recurrent, occurring between 14 and 90 days after diagnosis, was determined to be 18.1%. Interestingly, a 2-year retrospective cohort analysis involving 1,487,712 individuals diagnosed with COVID-19 revealed that the heightened incidence of mood and anxiety disorders in the context of COVID-19 was temporary and did not result in an overall surplus of these diagnoses when compared to other respiratory infections [112]. Conversely, the increased susceptibility to psychotic disorders, cognitive impairment, dementia, epilepsy, or seizures endured over the observation period. Individuals recovering from COVID-19 often experience PCS, most commonly characterised by chronic malaise, diffuse myalgia, depressive symptoms, and poor sleep quality [6, 113]. Cognitive impairment, often referred to as “cognitive fog”, is a common finding that affects memory, concentration, language, and executive function [6, 92, 114]. This cognitive decline is similar to what is observed in patients after critical illness, affecting around 20-40% of those discharged from the ICU [115].

Notable findings emerged in a comprehensive analysis of Patel *et al*. in 2022 involving 3,304 PCS patients in which a subset of individuals showed a plethora of symptoms including headaches (27.8%), fatigue (26.7%), muscle pain (23.14%), loss of smell (22.8%), altered taste (12.1%), sleep disturbances (63.1%), confusion (32.6%), focus impairment (22%), symptoms resembling PTSD (31%), feelings of depression (20%), and even thoughts of self-harm (2%) [116].

It is interesting to note that PCS and Chronic Fatigue Syndrome (CFS) have a significant overlap in their symptoms. Studies suggest that around 50% of PCS patients meet the diagnostic criteria for CFS six months after being infected with SARS-CoV2 [117, 118]. Both PCS and CFS are characterised by chronic pain and persistent fatigue that is not related to any musculoskeletal issues [119]. However, the exact cause of these symptoms remains unclear, and, like most pathophysiological mechanisms, the underlying mechanisms have yet to be confirmed.

In conclusion, SARS-CoV-2 is associated with a range of neuropsychiatric symptoms during acute infection, including delirium, depression, anxiety, memory impairment, and headaches. These symptoms can persist beyond the acute phase, with some patients experiencing ongoing cognitive issues, such as “brain fog,” and psychiatric conditions like depression and anxiety. PCS often includes chronic fatigue, myalgia, and sleep disturbances, with many symptoms overlapping with CFS. While the exact mechanisms behind these symptoms remain unclear, they highlight the significant and enduring impact of COVID-19 on mental health.

## 6 NAC AND ALC: OVERVIEW AND MECHANISM OF ACTION

N-Acetylcysteine (NAC), as an acetylated form of cysteine, markedly enhances bioavailability and stability, increasing its effectiveness in clinical and supplemental uses. Cysteine, a conditionally essential amino acid, is normally synthesised from methionine through a multi-step process, but its synthesis can be compromised under specific pathophysiological conditions [120].

NAC serves as a precursor to glutathione, a pivotal cellular antioxidant. NAC supports glutathione synthesis, enhancing detoxification and body antioxidant defences. Thus, its synthesis is crucial for maintaining cellular redox balance and is often limited by the availability of cysteine [121].

In clinical applications, NAC is used as a mucolytic agent for respiratory conditions, reducing mucus viscosity in the airways. Its hepatoprotective properties are notably significant in acetaminophen toxicity, where it reduces hepatic damage by replenishing intracellular glutathione levels [122].

NAC's properties are multifaceted, extending to neurodegeneration and inflammation treatment. Its precise mechanisms and clinical efficacy vary depending on the specific pathological condition and context [123-131].

NAC's substantial antioxidant properties stem from a complex array of biochemical pathways. It plays a critical role in neutralising free radicals and mitigating oxidative stress at the cellular level [132]. Additionally, NAC contributes to increasing levels of hydrogen sulfide and sulfane sulfur species, which have various signalling and regulatory roles in cells [133], and participates in reductant activities related to disulfide thiols, helping maintain protein structure and function under oxidative stress [134].

NAC also potentiates the entire glutathione enzymatic system, including glutathione-S-transferase (GST) and glutathione peroxidase (GPX), boosting the detoxification system [135]. It initiates the Nrf2 pathway, a critical regulatory pathway in cellular antioxidant defence, leading to the upregulation of various antioxidant genes [136]. Another aspect of NAC action is its modulation of nitric oxide metabolism, which is essential for vascular and neuronal functions [137].

The immunomodulatory properties of NAC are evident in its ability to reduce inflammatory cytokines such as TNF-α, IL-1β, NF-κB, IL-6, and IL-10, and to enhance CD4^+^ T cell counts [27, 124, 125, 127, 128].

Regarding neurotransmitter systems, NAC aids in the adjustment of group II metabotropic glutamate receptors (mGluRs), attenuating glutamatergic signalling. It enhances the endogenous activation of mGluR2 and mGluR3, promoting the expression of the EAAT2 [138]. NAC restores mGluR3 receptor activity through glutamate carboxypeptidase II (GCPII) inhibition, favouring the antioxidant response through Nrf-2 and Nf-κB pathways [139]. The enhancement of cognitive function is linked to changes in the kynurenine pathway within the DLPFC, affecting the interaction between the mGluR2 and 5-HT2A receptors and helping to prevent early stages of psychosis [27, 140]. NAC also activates the xCT transporter within astrocytes, increasing intracellular cysteine levels and facilitating glutathione synthesis, thereby modulating glutamate levels and NMDA receptor activity [129].

As a modulator of the kynurenine pathway, NAC reduces kynurenic acid levels *via* inhibition of the KAT/AadAT enzyme in the prefrontal cortex and influences dopamine release in the striatum, potentially through GSH-mediated modulation of glutamate receptors [23, 141]. The impact of NAC on dopamine release in animal models has been observed in the striatal nucleus and is linked to reduced dopamine transporter density in the striatum of rhesus monkeys [142]. The modulation of dopamine levels may occur through enhanced GSH production, impacting striatal dopamine levels *via* GSH influence on NMDA and non-NMDA glutamate receptors [143].

The cellular protective actions of NAC include activating ERK phosphorylation and MAP kinase-signaling pathways and protecting astrocytes against proteotoxic stress [144-149]. NAC extends its protective actions to glial and hippocampal cells, defending against oxidative stress-induced neuronal injury and preserving long-term potentiation [150, 151]. It also offers protection against endothelial damage *via* its nitric oxide (NO)-mediated vasodilatory activity [97].

NAC has therapeutic potential in treating neuropsychiatric diseases as an adjunctive treatment for disorders like obsessive-compulsive and related disorders, autism, major depression, bipolar depression, and schizophrenia [152]. It effectively treats chronic obstructive pulmonary disease (COPD) and pulmonary fibrosis [153]. NAC has been suggested as a treatment for COVID-19, reducing symptoms and hindering viral replication due to its thiol-based and hydroxide-rich properties [154]. It also shows promise in treating acute brain damage caused by COVID-19, serving as a potential adjunct therapy [155].

Acetyl-L-carnitine (ALC) is the acetylated version of L-carnitine, a naturally occurring amino acid derivative essential for energy production. This acetylation enhances ALC's ability to cross the blood-brain barrier, thus increasing its bioavailability in the central nervous system. ALC boosts mitochondrial metabolism and energy production by facilitating carnitine shuttling and fatty acids beta-oxidation [156-158]. Furthermore, it contributes to mitochondrial membrane stabilisation, leading to anti-apoptotic actions mainly through attenuating mitochondrial membrane peroxidation [158]. ALC also addresses carnitine deficiency in conditions such as CFS [159, 160].

In addition to its role in metabolism, ALC exhibits antioxidant and anti-inflammatory properties, which help reduce excessive immune responses [158, 161]. It also influences neurotransmitter release, exerting neurotrophic, neuromodulatory, and neuroprotective effects. ALC is known for its role in hippocampal neurogenesis and in the modulation of neurotrophic factors like NGF, GDNF, and BDNF in specific brain regions [158, 162-165]. Additionally, ALC increases acetylcholine synthesis by raising acetyl-CoA levels and enhancing choline acetyltransferase activity [162, 163].

ALC acts as an acetyl group donor, acetylating the Nf-kB p65 subunit and histone-3-lysine-27 (H3K27). This process affects the expression of GRM2, encoding for mGluR2, and plays a role in modulating glutamatergic signalling in the hippocampus and prefrontal cortex [165-170]. It also promotes the expression of the xCT subunit by acetylating REST in the xCT promoter [165, 171], which is crucial in hippocampal and DLPFC regions for enhancing NGF and BDNF activity [169].

Therapeutically, ALC is used in peripheral neuropathic pain, neurodegeneration, CFS, depression, dysthymic disorder, mitochondrial decay, and cognitive impairment [172-178]. Its epigenetic modulation of glutamatergic function is linked to its antidepressant, analgesic, and neurogenic properties [158, 163, 165, 169, 179-183]. ALC modulates various neurotransmitter systems, especially the cholinergic system, by providing acetyl groups for ACh synthesis [162, 167, 184].

ALC counteracts oxidative stress and senescence by up-regulating Heat-shock proteins, Nrf2 signalling, HO-1, and SOD and promotes the transcription of the Grm2 gene [158, 166, 167, 170, 185]. It affects brain iron homeostasis by potentially reversing iron-induced oxidative stress through down-regulation of ferritin-H gene expression, reducing intracellular iron reserves [185-187].

In COVID-19, fatty acid and carnitine metabolism alterations have been observed, predictive of disease severity [188]. ALC reduces pro-inflammatory cytokines and lowers inflammatory markers [161]. COVID-19 infection increases KYNA levels and GCPII protein expression in the brain, influencing glutamatergic dysfunction and neurotransmission in the DLPFC [23, 39, 94, 189-194]. The mGluR3 receptor, predominantly located in astrocytes, is involved in glutamate uptake and synaptic modulation, thus protecting against neuro excitotoxicity [69, 195, 196].

ALC's role as a transcriptional inducer of the mGluR2 gene in various brain regions highlights its potential to modulate glutamatergic activity [95, 165, 168-170]. Elevated extracellular glutamate levels contribute to the tonic activation of mGluR2 and mGluR3 receptors, which are crucial in synaptic glutamatergic modulation [197]. ALC has shown protective potential against severe COVID-19, post-COVID-19 fatigue, and relief from PCS symptoms [24, 198, 199].

Lastly, the interplay between Nf-κB and Nrf2 pathways significantly influences the progression of long-term COVID neuropsychiatric complications [200-202], and astrocyte-neuron communication *via* the astroglial xc-system is crucial for synaptic glutamatergic regulation [203].

Both NAC and ALC offer significant therapeutic potential due to their roles in enhancing antioxidant defenses, modulating neurotransmitter systems, and protecting against neurodegeneration. NAC's ability to replenish glutathione and reduce oxidative stress, along with ALC's support for mitochondrial function and neurotransmitter synthesis, make them valuable in treating a wide range of conditions, including neuropsychiatric disorders and complications related to COVID-19. Their multifaceted mechanisms of action highlight their importance in maintaining cellular health and combating inflammation and oxidative damage.

## 7 HYPOTHESIS AND RATIONALE OF NAC AND ALC IN NEUROPSYCHIATRIC MANIFESTATIONS OF ACUTE COVID-19 DISEASE, ONGOING SYMPTOMATIC COVID-19, PCS

NAC and ALC hold promise as therapeutic agents for neuropsychiatric conditions associated with COVID-19 and PCS [23, 24, 204]. They exhibit a multifaceted mechanism of action, addressing oxidative stress, neurotransmitter imbalances, and neuroinflammation through Nf-κB inhibition, replenishing GSH reserves, and alleviating the impairment of mGluR2 and mGluR3 [136, 205], acting on inflammatory state, lowering serum inflammatory cytokines and markers (IL-6, TNF-alfa, PCR) [161].

As stated above, according to several evidence, acute COVID-19 damage to the CNS may play a central role in the pathogenesis of neuropsychiatric symptoms through several pathophysiological mechanisms: dysregulation of inflammation, astrocytic glial damage, glutamatergic impairment (shift in tryptophan metabolism towards kynurenine pathway, Nf-κB, oxidative stress, glutamatergic and excitotoxic damage) and possible glymphatic impairment [15, 46, 82]. Furthermore, Tavares-Júnior *et al*. (2022) suggest that memory impairment, resulting from acute phase alterations in the hippocampus, may lead to persistent neurological complications in PCS [206]. Moreover, it has been demonstrated that even mild COVID-19 inflammation or infection, not directly involving the brain, can lead to neuroinflammation and long-term cognitive impairment [81]. Wang highlighted the role of the mGluR2-TfR1 axis as a potential pathway for neurotropic viruses and their role in ferroptosis [38].

NAC and ALC's role in treating psychiatric conditions, such as mood disorders and chronic pain due to their glutamatergic modulatory activity, has been supported by multiple studies (Mohiuddin *et al*., 2021; Morris *et al*., 2019; Ooi *et al*., 2018; Sarzi-Puttini *et al*., 2021; Vermeulen and Scholte, 2004; Wang *et al*., 2014) [207-210]. Their potential in mitigating COVID-19-associated neuropsychiatric symptoms and PCS is promising, as evidenced by several researchers [24-28].

Preliminary studies have shown that a combination of supplements, including NAC and ALC, significantly reduces recovery time in patients with mild to moderate COVID-19 [211]. These findings align with the metabolic changes observed in COVID-19 patients [212]. Some authors recently suggested that long-term COVID-related cognitive dysfunction might improve with the intake of NAC and guanfacine [28]. Moreover, Scaturro *et al*., 2022 and colleagues highlighted the role of ALC in treating depressive symptoms associated with Long COVID [24].

The metabolic changes in COVID-19 highlight the suitability of NAC and ALC for their antioxidant properties and ability to compensate for metabolic alterations, such as replenishing GSH reserves or carnitine supplementation [213-216]. A negative correlation between serum carnitine levels and COVID-19 susceptibility and severity has been described [188].

NAC and ALC have a synergistic effect in modulating glutamatergic activity on the xc-/mGluR2 network through epigenetic mechanisms [165]. This synergy extends to neuron and astrocyte protection. They also mitigate Nf-κB overactivated signalling, attenuating the NLRP3 inflammasome pathway and decreasing pro-inflammatory cytokines and PCR levels, thereby showcasing their immunomodulatory activities [161, 202, 217]. Their efficacy in protecting against cytokine storms has also been noted [82, 135, 218].

NAC, as a substrate precursor of the xc- system, normalises DLPFC neurotransmission and may enhance mGluR3 signalling, improving DLPFC network connectivity and neuronal firing [141, 197, 203, 219]. Additionally, ALC has been found to ameliorate chronic fatigue and tiredness through intracellular mechanisms [199, 204, 220].

In addition, NAC thiols play a crucial role by effectively obstructing the ACE2 receptor, impeding the cellular penetration of SARS-CoV-2 [27]. An *in vitro* study has shown that L-carnitine treatment may mitigate SARS-CoV-2 infection [221]. Interestingly, the administration of NAC determines an augmentation in endogenous hydrogen sulfide production, which has an anti-viral host factor role [222, 223]. SARS-CoV-2 infection can induce death or damage to brain endothelial cells through SARS-CoV-2 protease, which cleaves the Nf-κB essential modulator (NEMO), leading to BBB disruption and subsequent neuroinflammation [102]. The hyperactivated Nf-kB signal in the CNS induces reactive changes in microglia and astrocytes, further amplifying the neuroinflammatory response [15, 224]. Another noteworthy aspect of NAC is its potential contribution to preventing and controlling infections caused by RNA viruses [225] by enhancing the signalling functions of TLR7 and the mitochondrial antiviral signalling protein, resulting in increased production of IFN-1 [226]. Moreover, ALC and NAC mitigate Nf-kB overactivated signal, attenuating NLRP3 inflammasome pathway (IL1β and IL18) and decreasing serum TNF-alpha, IL-1, IL-6 levels, and PCR [161, 202, 217], thereby their immunomodulatory activities [161, 227, 228], and protecting against cytokine storms [82, 135, 218].

Interestingly, ALC is a transcriptional inducer of the mGluR2 gene in the prefrontal cortex, hippocampus, and spinal circuits, thus modulating glutamatergic activity [95, 165, 168, 169]. Once neuroinflammation is established, the glutamatergic neurotransmission is impaired [15]. Nf-κB signal negatively regulates EAAT2 expression, which is crucial for astrocyte neuroprotection [229]. Astrocyte-neuron communication is essential for synaptic glutamatergic modulation, which uses the astroglial xc-system, which extrudes intracellular glutamate in exchange for extracellular cysteine [203]. This system is functionally connected to the mGluR2 of the presynaptic neuron and mediates important negative feedback, providing dynamic regulation of extracellular glutamate levels [197]. Elevated extracellular glutamate levels contribute to the tonic activation of mGluR2 and mGluR3, predominantly located presynaptically [69]. Therefore, this modulation is essential for mitigating the risk of excitotoxicity [196]. Moreover, the COVID-19 condition is associated with increased GCPII expression in astrocytes [28, 192] and a shift in tryptophan metabolism toward the kynurenine pathway (Boldrini *et al*., 2021), resulting in altered mGluR3 and NMDA receptor signalling [28]. Mainly, ALC and NAC have synergistic glutamatergic modulatory activity on the xc-/mGlu2 network *via* epigenetic mechanisms [165], exerting neuron and astrocyte protective effects. NAC is a substrate precursor of the xc- system [197, 203]. It may normalise DLPFC neurotransmission and mediate procognitive action by blocking the production of KYNA in DLPFC [141] and may enhance mGluR3 signalling [219], which ameliorates DLPFC network connectivity and DLPFC neuronal firing [28]. It also can exert modulatory effects on the kynurenine pathway [141]. ALC ameliorates chronic fatigue and tiredness through intracellular mechanisms [199, 204, 220]. Moreover, the metabolic changes described in COVID-19 make ALC and NAC particularly suitable for their well-known antioxidant properties and their ability to compensate local and systemic for metabolic established secondary to this pathological condition [213-216]. Interestingly, a negative correlation has been recently described between serum carnitine levels and COVID-19 susceptibility and severity [188].

In the context of COVID-19 pathology, neuropsychiatric manifestations, and PCS, mGluR2 could be viewed as a mediator that links the continuum between nociception, pain, negative mood, and depression [230]. Interestingly, in the context of SARS-CoV-2 infection, alterations in lipid metabolism and the xc-GSH-GPX4 axis, pivotal mechanisms of ferroptosis, are involved [231, 232], and further components of this mechanism, such as TfR1 and mGluR2, are crucial for the virus's internalisation, making it a potential therapeutic target.

Moreover, it is possible to hypothesise subsequent down-regulation of neuronal mGluR2, as already demonstrated for ACE2 [233]. In particular, xCT and mGluR2 downregulation at the hippocampal level correlates with depressive and low resilience phenotypes to stress [165], and the reduced expression of mGluR2 may provide insights into the virus-induced vulnerability to stress-related conditions [20, 168]. Thus, understanding the mechanisms of internalisation and whether this process involves heterodimeric receptors (Wang *et al*., 2023) would be intriguing [234].

**Fig. (3):**
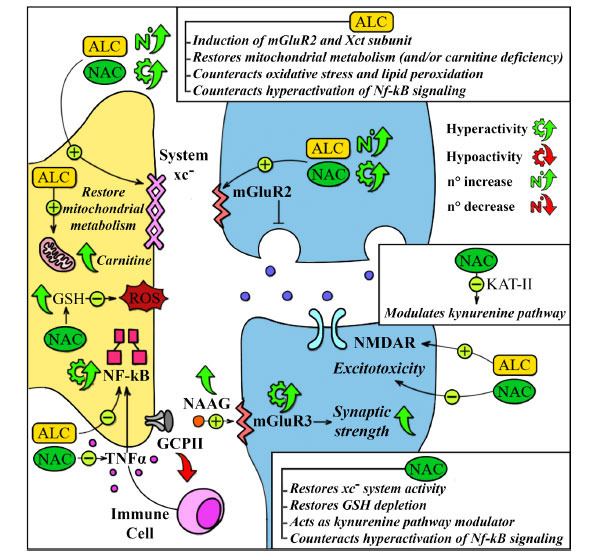
Potential pathophysiological mechanisms and the synergistic effect of NAC and ALC. **Abbreviations:** NAC: N-Acetyl-Cysteine; ALC: Acetyl-L-Carnitine; ROS: Reactive oxygen species; GSH: Glutathione; EAAT: excitatory amino acid transporter; NF-kB: Nuclear Factor-kappa B; NAAG: N-Acetyl-Aspartyl-Glutamate; mGluR3: metabotropic Glutamate 3 receptor; mGluR2: metabotropic Glutamate 2 receptor; Glu: Glutamate; Gln: Glutamine; Cys: Cysteine; System xc^-^: cystine/glutamate antiporter system; Xct subunit (SLC7A11): light-chain subunit of cysteine/glutamate antiporter; GCPII: Glutame Carboxypeptidase II; NMDAR: N-methyl-D-aspartate Receptor; TNFα: Tumor Necrosis Factor α; KAT-II: kynurenin aminotransferase II.

Ferroptosis also appears to be an intersection involving iron homeostasis, lipid metabolism, and the endogenous antioxidant system xc-GSH-GPX4. In particular, viral infections, such as SARS-CoV-2 infection, seem to play a significant role in influencing the latter [231, 232]. ALC, by acting on the lipid peroxidation system, and NAC, by supplying the limiting factor in the pathway, cysteine, seem to be particularly well-suited for addressing this context [235]. ALC and NAC induce a rapid antidepressant and pro-resilience response by stimulating specific hippocampal areas [165]. NAC and ALC are also suggested to have neuroprotective functions, particularly on astrocytes, potentially reducing glymphatic impairment and improving symptoms or preventing neuropsychiatric manifestations of PCS [236] (Fig. **3**).

The clinical efficacy of ALC and NAC has been evaluated in several trials [167, 172, 176, 199, 210, 237], the preliminary encouraging data and physiopathological frameworks support the need to study these compounds in this context and clarify their role [23, 24, 28, 199].

Most studies have demonstrated that in conditions of carnitine deficiency and inflammation, ALC dosages between 1500-2000 mg/day can be effective in treating fatigue and improving immune response [160, 204, 214, 238]. In conditions characterised by high oxidative stress and inflammation, such as psychiatric disorders, the dosages of NAC of 2000-2400 mg/day are recommended [207]. In a small study of 12 patients with long-term COVID-related cognitive dysfunction conducted by Fesharaki *et al*. (2022), a dosage of 600 mg NAC daily was used [28]. In a randomised study by Scaturro *et al*. (2022), ALC was administered with a dosage ranging from a minimum of 500 mg per os to 1000 mg per day with intramuscular injection [24].

Therefore, in the light of dosing under conditions of high oxidative stress, inflammation, and carnitine deficiency, considering recent results in PCS, it seems reasonable to recommend a dosage of ALC and NAC between 1500-2000 mg and 2000-2400 mg per day, respectively. It might be interesting to use such compounds in clinical practice as a supplement, especially as an add-on to symptom-dependent drug therapy, albeit with due caution.

## CONCLUSION

This review aimed to elucidate the principal mechanisms involved in the neuropsychiatric manifestations of COVID-19, which involve glutamate dysregulation, Xct, Nf-κB, mGluR2, and mGluR3, astrocyte and glial damage and neurotoxicity highlighted as central factors [15], exploring the potential of NAC and ALC as promising therapeutic agents in neuropsychiatric conditions COVID-19-related, including those associated with PCS.

Furthermore, the elucidation of the viral entry mechanism through mGluR2, due also to its predominant expression in brain tissues, unlike the recognised internalising receptor ACE2, may provide insights into the severe impact of COVID-19 on the brain and its intricate relationship with astrocyte damage, aberrant neuroinflammation, excitotoxicity, impairment of the glymphatic system as well as DLPFC and hippocampal injury [18, 233, 239].

The provided evidence and well-conducted clinical trials could pave the way for exploiting these supplements as a treatment for COVID-19-related neuropsychiatric symptoms and PCS. This approach holds the potential for preventing both short and long-term brain damage, with a specific emphasis on vulnerable areas like the CA1 and CA3 regions, vDG of the hippocampus, as well as the DLPFC [86, 92, 93, 240-242].

In this context, we postulate that NAC and ALC could operate on several levels to manage Neuropsychiatric manifestations of COVID-19 disease and PCS.

- They serve as a first-line defense by preventing SARS-CoV-2 infection in the nasal mucosa, countering Nf-kB activity and its over-activated signalling pathway, and counteracting virion binding.
- Once the acute inflammatory state is established, NAC and ALC help modulate inflammatory activity in the CNS at systemic and local levels.
- After neuroinflammation, NAC could 'restore' proper mGluR3 signalling, thereby inducing the expression of astrocytic EAAT-1 and EAAT-2. It also plays a combined action with the ALC role in 'restoring' the xc-/mGluR2 system, reinstating adequate astrocyte-neuron communication, ultimately mitigating neuro excitotoxicity.
- Once oxidative stress is established, NAC is a substrate that restores the GSH pool and functions as an antioxidant agent. ALC counteracts oxidative stress and provides anti-apoptotic effects.
- NAC reduces KYNA levels, potentially ameliorating COVID-related cognitive dysfunction (with a procognitive effect in Prefrontal circuitries).
- ALC and NAC could ameliorate fatigue through different mechanisms of action, including combined action on glutamatergic neurotransmission.

In conclusion, this work emphasises the complex interplay of various factors in the neuropsychiatric manifestations associated with COVID-19, highlighting the importance of glial cells, glutamate dysregulation, glymphatic system alterations, and NAC and ALC's potential therapeutic role. It calls for further research to unveil the intricate mechanisms at play and explore novel avenues for treatment and prevention. Furthermore, the clinical interest in these compounds stems from the lack of standardised pharmacological treatment for PCS and related COVID-19 neuropsychiatric symptoms. However, despite the evidence provided in this review, it is necessary to consider the speculative nature of our inferences.

## LIST OF ABBREVIATIONS

ACE2: Angiotensin-converting Enzyme 2 Receptor
ARDS: Acute Respiratory Distress Syndrome
BBB: Blood-brain Barrier
BCB: Blood-cerebrospinal Fluid Barrier
BDNF: Brain-derived Neurotrophic Factor
cAMP: cyclic Adenosine Mono-phosphate
CFS: Chronic Fatigue Syndrome
CNS: Central Nervous System
CVOs: Circum Ventricular Organs
DLPFC: Dorso-Lateral Prefrontal Cortex
DPP4: Dipeptidyl-peptidase 4 Receptor
EAAT2: Excitatory Amino-acid Transporter 2
GCPII: Glutamate Carboxypeptidase II
GDNF: Glial Cell-derived Neurotrophic Factor
GFAP: Glial Fibrillary Acidic Protein
GSH: Glutathione
IFN: Interferon
IL: Interleukin
KYNA: Kynurenic Acid
MCP: Monocyte Chemoattractant Protein
mGlu: Metabotropic Glutamate Receptor
MODS: Multiple Organ Dysfunction Syndrome
NAC: N-Acetyl Cysteine
NEMO: Nf-kB Essential Modulator
Nf-kB: Nuclear Factor Kb
NGF: Nerve Growth Factor
NICE: National Institute for Care Excellence
NMDA: N-methyl-D-aspartate Receptor
Nrf2: Nuclear Factor Erythroid 2-related Factor
PCS: Post-Covid Syndrome
PTSD: Post-traumatic Stress Disorder
REST: Repressor Element one Silencing Transcription Factor
RNS: Reactive Nitrogen Species
ROS: Reactive Oxygen Species
TfR1: Transferrin Receptor
TLR7: Toll-like Receptor 7
TNF: Tumoral Necrosis Factor
Xct: Cystine-glutamate Antiporter
